# How institutional support enhances teacher engagement in online teaching: chain mediation effects of digital self-efficacy and negative emotions

**DOI:** 10.3389/fpsyg.2025.1601764

**Published:** 2025-07-22

**Authors:** Dongping Liu, Zhonghua Sun, Yulu Cui

**Affiliations:** ^1^School of Education, Changchun Normal University, Changchun, Jilin, China; ^2^School of Educational Science, Xinyang Normal University, Xinyang, Henan, China

**Keywords:** institutional support, teacher work engagement, digital self-efficacy, negative emotions, online teaching

## Abstract

Online teaching has become a cornerstone of educational continuity in the era of digital education. However, sustaining teacher engagement in online teaching persists as a critical barrier to teacher sustainability. This study aims to investigate how institutional support enhances teachers’ work engagement through digital self-efficacy and negative emotions in online teaching. Participants were 232 K-12 teachers from different schools in China. A structural equation modeling (SEM) was used to confirm the initial model hypotheses regarding the relationship between variables. The findings show that institutional support is positively related to teacher work engagement, with digital self-efficacy and negative emotions as significant mediators. Finally, implications for educational administrators and teachers were discussed, with a focus on the importance of focused interventions that address teachers’ emotional and technological needs to improve their work engagement in online teaching.

## 1 Introduction

Digital technology has become indispensable in modern education, enabling new teaching models that enhance communication and resource accessibility for teachers and students (Balalle, 2024; Wang et al., 2024; Bond et al., 2020). The COVID-19 pandemic starkly demonstrated this necessity, as schools globally shifted abruptly to online instruction during lockdowns (Howard et al., 2021). It has resulted in a transition from face-to-face instruction to large-scale online teaching and learning (Tartavulea et al., 2020). By 2023, over 85% of K-12 teachers in China reported using online platforms for daily instruction, reflecting a nationwide push toward digital education (Ministry of Education of China, 2023). Teaching is a massive workload, made more difficult by the rapid shift to online teaching (MacIntyre et al., 2020). This transition exposed systemic vulnerabilities in educational preparedness, with teachers facing unprecedented workloads and psychological strain (Kim and Asbury, 2020). While digital tools offer solutions, their integration demands more than technical skills—teachers must also navigate emotional challenges and institutional barriers to sustain engagement (Scherer et al., 2021).

For professional growth to take place and for teaching effectiveness to increase, teachers must be sufficiently engaged in their work (Sánchez-Cabrero et al., 2021). Research underscores the effectiveness of online teaching is impacted by teachers’ work engagement (Ou et al., 2023). Highly engaged teachers correlate with improved student learning outcomes, including satisfaction (Martin et al., 2022), achievement (Hoque et al., 2023), and engagement (Wang et al., 2022). Conversely, low engagement exacerbates attrition risks, reduces job satisfaction (Ji and Zhao, 2020), increases intention to leave the job (Guo et al., 2021). These issues are amplified in online contexts, where isolation and technostress further strain educators (Poulou and Garner, 2024; Zhao et al., 2022). Therefore, teacher work engagement is key to the sustainability of high-quality online teaching (Zuo et al., 2024). Recent studies have highlighted the significance of support for innovation, school support, or institutional support in fostering a conducive environment for teacher work engagement (Joo et al., 2016). According to the job demands-resources (JD-R) theory, job resources such as institutional support have significant effects on teachers’ work-related emotions, which are predictors of work engagement (Bakker and Demerouti, 2017). This support plays an important role in instilling a sense of value and security in teachers, which improves their work engagement. Teachers who perceive great support from their institutions are more likely to experience positive emotions, self-efficacy, and a higher level of autonomous motivation, which leads to increased work engagement (Zhang et al., 2021).

Despite growing recognition of these challenges, two gaps persist. First, most studies focus on traditional classroom settings, neglecting the unique dynamics of online teaching (Wang et al., 2022). Second, while institutional support is acknowledged as vital (Tuiloma et al., 2022), the mechanisms for enhancing engagement through emotional regulation and digital self-efficacy remain underexplored. Addressing these gaps, this study investigates how institutional support fosters teacher engagement in online environments by simultaneously mitigating negative emotions and strengthening digital self-efficacy. It hopes to provide insights into the factors that contribute to teacher work engagement and effectiveness in the digital teaching environment, offering practical implications for teacher training and support initiatives.

## 2 Literature review and hypotheses

### 2.1 Work engagement in online teaching

“Teaching engagement” is derived from ‘work engagement’. Kahn (1990) defines work engagement as “*the control of an organization member’s self over his or her work role*” and the use and expression of energy, cognition, and emotion in the workplace. Schaufeli et al. (2002) defined ‘work engagement’ as “*a positive, work-related emotional and cognitive state, included three core dimensions: vigor, dedication, and absorption*.” He also noted that the three core components correlate to Kahn’s proposed energy, emotion, and cognition. This view was widely recognized (Salanova and Schaufeli, 2009).

This study defines online teaching engagement as “the sum of energy, emotion, and cognition that teachers invest in online teaching” based on the definition of Schaufeli et al. It includes the three characteristics of vigor, dedication and concentration. Studies have also demonstrated that work engagement is associated with positive outcomes at individual and organizational levels, such as higher work performance, higher health level, and higher organizational commitment goals (Innstrand et al., 2012; Bakker and Demerouti, 2008).

### 2.2 Institutional support for online teaching

Terms like “school support,” “support for innovation” and “institutional support” are frequently used when analyzing the integration of technology into teaching. This study employs the term “institutional support.” The institutional support examined in this study is based on Moreira-Fontán et al.’s (2019) definition as teachers’ perceived school support for innovation and the use of information technology. In the context of online teaching, when information technology is fully used, this concept has two dimensions. First, support for innovation and creativity, as well as the use of innovative teaching methods. Second, support for the use of technology in online teaching.

Institutional support is a type of job resource. Job Demands-Resources Theory hypothesizes that job resources reduce the effects of job demands on stress, including job anxiety (Bakker and Demerouti, 2017). Job resources are also a relevant predictor of work engagement. Some studies have provided additional evidence for this interaction. Guglielmi et al. (2016) found a positive correlation between teachers’ job resources and work engagement. There is a motivational connection between the employee and the workplace that triggers a higher level of work engagement (Sortheix et al., 2013). A study of home care professionals found that some job resources can slow down job demands and burnout (Xanthopoulou et al., 2007). Furthermore, institutional support was found to be a negative predictor of teachers’ ICT stress but a positive predictor of teachers’ positive views, attitudes toward ICT (Joo et al., 2016), and self-efficacy in teaching using technology (Admiraal et al., 2017). School support for innovation provides teachers with psychological comfort. The higher the teachers’ satisfaction with the support, the more positive their attitudes toward teaching (Cross and Hong, 2012). Therefore, this study proposes the hypothesis 1.

*Hypothesis 1*: There is a significant positive effect of institutional support on work engagement in online teaching (H1a). There is a significant positive effect of institutional support on digital self-efficacy (H1b). There is a significant negative effect of institutional support on negative emotions (H1c).

### 2.3 Teachers’ negative emotions in online teaching

Teachers’ emotions play a major role in their lives. In addition to being crucial for teachers’ mental health, it is strongly correlated with teaching behaviors, teacher-student relationships, and student outcomes in the teaching and learning process (Schutz and Zembylas, 2009). According to Farouk (2012), teachers’ emotions are essential to their interactions with students, colleagues, and parents rather than being “inner feelings” that stay fixed within the body. It is defined as a person’s mental activity, comprehension of other people’s emotions, capacity for emotion regulation, and reaction to emotional activity. Online teaching is a process of interaction with others. Due to various uncertainties in the teaching environment, teachers may experience both positive and negative emotions during this process. According to this study, teachers’ emotions during online teaching are defined as temporary, dynamic, and interactive emotional states brought on by a variety of uncertainties in online teaching. Additionally, it refers to the psychological experience of attitudes toward individuals, things, and objects that are associated with online teaching, including both positive and negative emotions. Negative emotions related to online teaching can be caused by a variety of factors, such as teachers’ lack of expertise in it, the digital environment, and their low level of digital literacy. Happiness, anger, and anxiety are the three emotions most closely associated with teaching; happiness is the most positive emotion, anger is the most negative emotion, and anxiety is the most interesting to researchers (Frenzel et al., 2016). Teachers experience anger at least once in about 15–20% of their classrooms. Anxiety is also one of the most concerned emotions related to the use of technology, which can prevent teachers from using technology (Joo et al., 2016).

Emotion plays a crucial role in all teaching activities of technology applications (Azzaro and Martínez Agudo, 2018). Wang (2014) emphasized the importance of emotions and emotional awareness in teaching and learning when using technology. Massive online teaching is the most powerful sign of technology-enabled teaching and learning. In this context, teacher emotions would have an impact on teachers’ confidence and intention to use technology, as well as the teaching and learning process. Technology-related anxiety or fear among teachers is caused by the unconsidered use of technology in the teaching and learning process, which hinders learning (Oluwalola, 2015). Ultimately, this has a negative impact on the effective application of technology. Perceived ease of use of technology is positively connected with pre-service teachers’ enjoyment (Teo and Noyes, 2011). In addition, pre-service teachers’ anxiety was negatively correlated with their technology knowledge (Kay, 2008).

However, negative emotions have been neglected in studies of teacher work engagement. Since work engagement has been defined as a positive work-related affective-motivational state (Schaufeli et al., 2002), it is important to explore the relationship between teachers’ negative emotions and work engagement in online teaching. Schaufeli et al. (2001) concluded that employees with high work engagement have higher positive emotions and lower negative emotions. The study of novice teachers’ anxiety found that there is a negative relationship between teachers’ work engagement and anxiety (Zhang, 2010). Therefore, hypothesis 2 was proposed in this study.

*Hypothesis 2*: Teachers' negative emotions have a significant negative effect on work engagement in online teaching (H2a). Teachers' negative emotions would mediate the effects of institutional support on work engagement in online teaching (H2b).

### 2.4 Digital self-efficacy for online teaching

Digital self-efficacy (DSE) in online teaching contexts in this study is defined as *teachers’ domain-specific confidence in their ability to effectively integrate digital technologies into pedagogical practices, encompassing both technological operation and instructional design capacities.* This concept originates from Bandura’s (1977) self-efficacy theory but is contextualized to digital education, emphasizing dual competencies in technical operation and instructional strategies. And according to Moreira-Fontán et al. (2019), there are two components of digital self-efficacy in teaching. One is digital technology competency, which includes technological knowledge and the capacity to use digital technology resources (e.g., online platforms, multimedia resources, data analytics software). The other is pedagogical competence, which can also be considered content knowledge or pedagogical knowledge. Encompasses the capacity to translate technology into pedagogical resources, such as designing interactive online activities, leveraging data-driven feedback to optimize instruction, and sustaining student cognitive engagement in digital environments. This construct diverges from general self-efficacy in three critical dimensions: (a) task specificity (focused on technology-mediated teaching scenarios rather than general life challenges) 3; (b) competency duality (requiring simultaneous mastery of technological tools and pedagogical principles) (Ulfert-Blank and Schmidt, 2022); and (c) dynamic responsiveness (adapting to rapidly evolving digital ecosystems) (Hwang et al., 2023).

In addition, the concepts of technological competence and digital self-efficacy are also different. Although technological competence focuses on objective skill acquisition (e.g., coding, software operation), digital self-efficacy incorporates individual beliefs. For instance, two teachers with equivalent technical skills may exhibit divergent innovation intentions in online teaching due to differences in self-efficacy (Sehar and Alwi, 2023). Empirical evidence suggests that technological competence forms the foundation of digital self-efficacy, but the latter also requires synergistic support from teaching experience and institutional support (e.g., training, peer collaboration) (Hatlevik and Hatlevik, 2018).

With the development and use of technology, there are many studies on computer self-efficacy (Hatlevik et al., 2018; Wartella and Jennings, 2000) and Internet self-efficacy (Chang et al., 2014; Joo et al., 2000). Digital self-efficacy is associated with learning and developing new skills and also influences learners’ willingness to engage in digital systems (Ulfert-Blank and Schmidt, 2022).

Based on the JDR theory, self-efficacy is considered one of the main personal resources that predicts work engagement. A study showed that a higher level of personal resources, including self-efficacy, correlates with higher levels of work engagement (Xanthopoulou et al., 2009). People with low self-efficacy would experience more negative emotions (Schwarzer and Hallum, 2008). Research suggests that self-efficacy for teaching correlates positively with positive emotions and negatively with negative emotions (Taxer and Frenzel, 2015). Additionally, Moreira-Fontán et al. (2019) showed that teachers’ ICT-related positive emotions mediate the effects of digital self-efficacy on work engagement and that teachers’ digital self-efficacy positively predicts teachers’ ICT-related positive emotions. Therefore, the following hypothesis 3 was proposed in this study.

*Hypothesis 3*: Digital self-efficacy has a significant positive effect on work engagement (H3a). Digital self-efficacy has a significant negative effect on online teaching negative emotions (H3b). Digital self-efficacy mediates the effects of institutional support on work engagement in online teaching (H3c). Digital self-efficacy and negative online teaching emotions would mediate the effects of institutional support on work engagement in online teaching (H3d).

**Figure 1:**
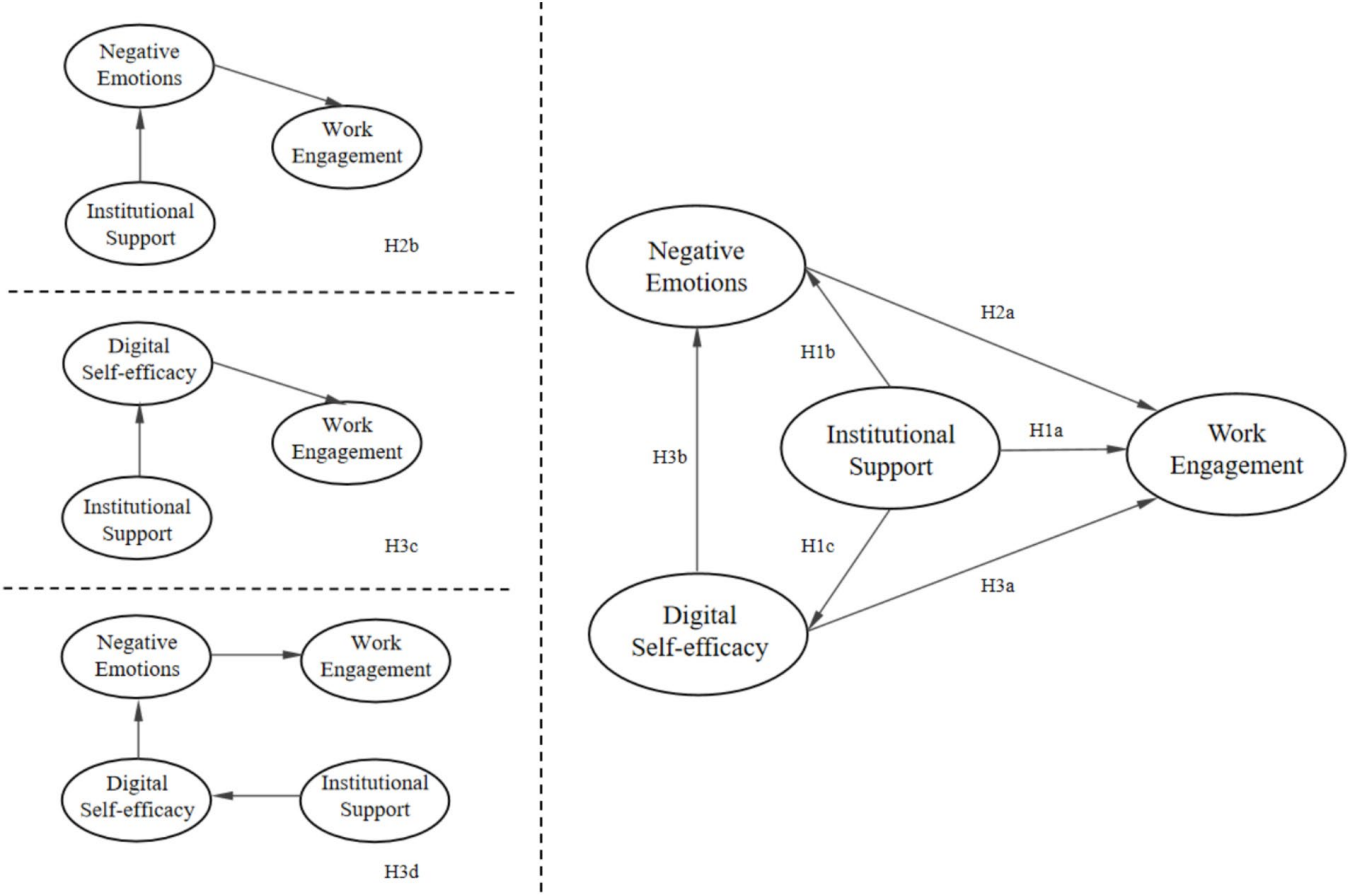
Proposed model of relations between variables.

Drawing from previous research, this study aims to analyze the structural relationships among four variables: work engagement, institutional support, negative emotions, and digital self-efficacy. As shown in Figure 1, six hypotheses of direct relationship and three hypotheses of mediated relationships were proposed.

## 3 Methods

### 3.1 Participants

In total, 246 teachers were contacted. They were from different primary and secondary schools in China. All the participants had experience with online teaching. After the removal of incomplete and invalid questionnaire responses. There were 232 valid responses. The specific statistics are shown in Table 1.

**Table 1:** Sample information.

Measured feature		Numbers	%
Gender	Male	70	30.2
	Female	162	69.8
Age	20–29	114	49.1
	30–39	74	31.9
	40–49	35	15.1
	≥50	9	3.9
Teaching year	0–5	139	59.8
	6–10	36	15.7
	>10	57	24.5
Education level	associate degree	6	2.6
	undergraduate degree	42	18.0
	graduate degree	184	79.4

### 3.2 Instruments

The questionnaire included two parts. The first part was socio-demographic variables, such as gender, age, teaching year, and educational level. The second part was target variables. And the items were presented.

#### 3.2.1 Work engagement

The Utrecht Work involvement Scale (UWES-9), developed by Schaufeli et al. (2006), was used to assess teachers work engagement in online teaching. It consists of three dimensions: vigor, dedication, and absorption, with three items for each dimension, totaling 9 items (Vigor: e.g. “In online teaching, I feel bursting with energy”; Dedication: e.g. “I find online teaching full of meaning and purpose”; Absorption: e.g. “I am immersed in online teaching”). A 7-point Likert scale was used, ranging from 0 (strongly disagree) to 6 (strongly agree). In this study, the Cronbach’s alpha of the scale was 0.893.

#### 3.2.2 Institutional support

To assess institutional support for online teaching provided by the school, the scale of Innovative Climate was applied (De Pablos et al., 2011; Moreira-Fontán et al., 2019). This 3-item instrument assesses the school’s level of receptivity, creativity stimulation, and innovation in general (e.g., “Our school supports and encourages innovation, such as online teaching”). The three items adopted a 5-point Likert-type scale, and it is easy to understand the complete list of scale descriptors (1 = “strongly disagree,” 2 = “disagree,” 3 = “neither disagree nor agree,” 4 = “agree,” and 5 = “strongly agree”). In this study, the Cronbach’s alpha of the scale was 0.813.

#### 3.2.3 Teachers’ negative emotions

Teachers’ negative emotions in online teaching were assessed by adopting the 8-item Teachers’ Emotion Scale (TES) developed by Frenzel et al. (2016). It includes the anxiety dimension (4 items; e.g., “I am often worried that my online teaching is not going so well.”) and the anger dimension (4 items; e.g., “I often have reason to be angry while I teach these students in online teaching”). A 4-point Likert scale was adopted, ranging from 1(strongly disagree) to 4(strongly agree). In this study, the Cronbach’s alpha value of the scale was 0.899.

#### 3.2.4 Digital self-efficacy

The scale of Teachers’ Knowledge about technology and the internet (Sigalés et al., 2008) was applied to evaluate teachers’ digital self-efficacy for online teaching. It consists of 3 items (e.g., “I can effectively explain or demonstrate online by using digital technology”). A four-point Likert scale was used with the options of “strongly agree,” “agree,” “disagree,” and “strongly disagree” and was scored as 4, 3, 2, and 1. In this study, the Cronbach’s alpha of the scale was 0.876.

### 3.3 Data analysis

**Figure 2:**
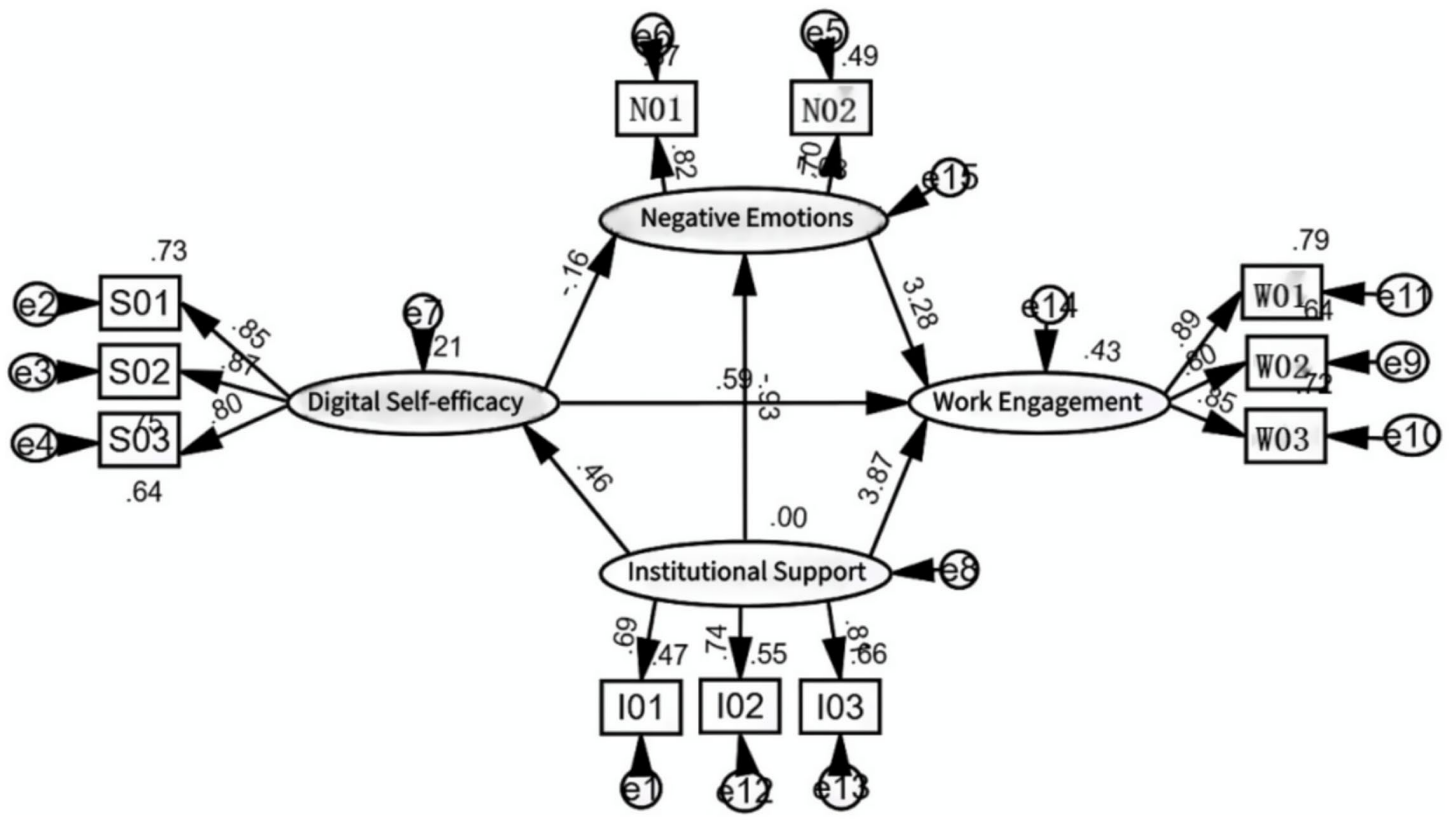
The measurement structural model (standardized parameter estimates).

The data analysis was conducted with a three-step methodology. In the first step, the reliability and validity of the measurement model were tested by Cronbach’s alpha (Cronbach’s *α*) and confirmatory factor analysis (CFA) using SPSS19.0 software. In the second step, a t-test and analysis of variance (ANOVA) were conducted to investigate whether gender and teaching age were related to work engagement, institutional support, teachers’ negative emotions and digital self-efficacy. In the third step, the research model (Figure 2) was analyzed using structure equation modeling (SEM), supported by AMOS 26.0. The paths between the four variables were modified.

## 4 Results

### 4.1 Measurement model testing

Cronbach’s α was calculated to validate the internal consistency reliability. The value of Cronbach’s α ranged from 0.813 to.899. Additionally, the validity was tested through confirmatory factor analysis (CFA). The meanings of all the items were clearly described and distinguished from one another. All the items included in the four common variables were consistent with the classification.

The potential relationships were tested by SEM. AMOS 26.0 software was used to test the hypothesized model. The measurement model is shown in Figure 2. The measurement model tests of goodness of fit were a prerequisite condition for valid interpretations of the variables in structural relationships (Kline, 2005). As shown in Table 2, the measurement model needed to be modified to satisfy goodness of fit. Therefore, the measurement model was modified twice based on data and hypotheses.

**Table 2:** Measurement model fit index.

	χ2 /df	AGFI	GFI	NFI	CFI	IFI	RMSEA
Values	3.606	0.869	0.912	0.917	0.938	0.938	0.108

### 4.2 Structural equation model

**Figure 3:**
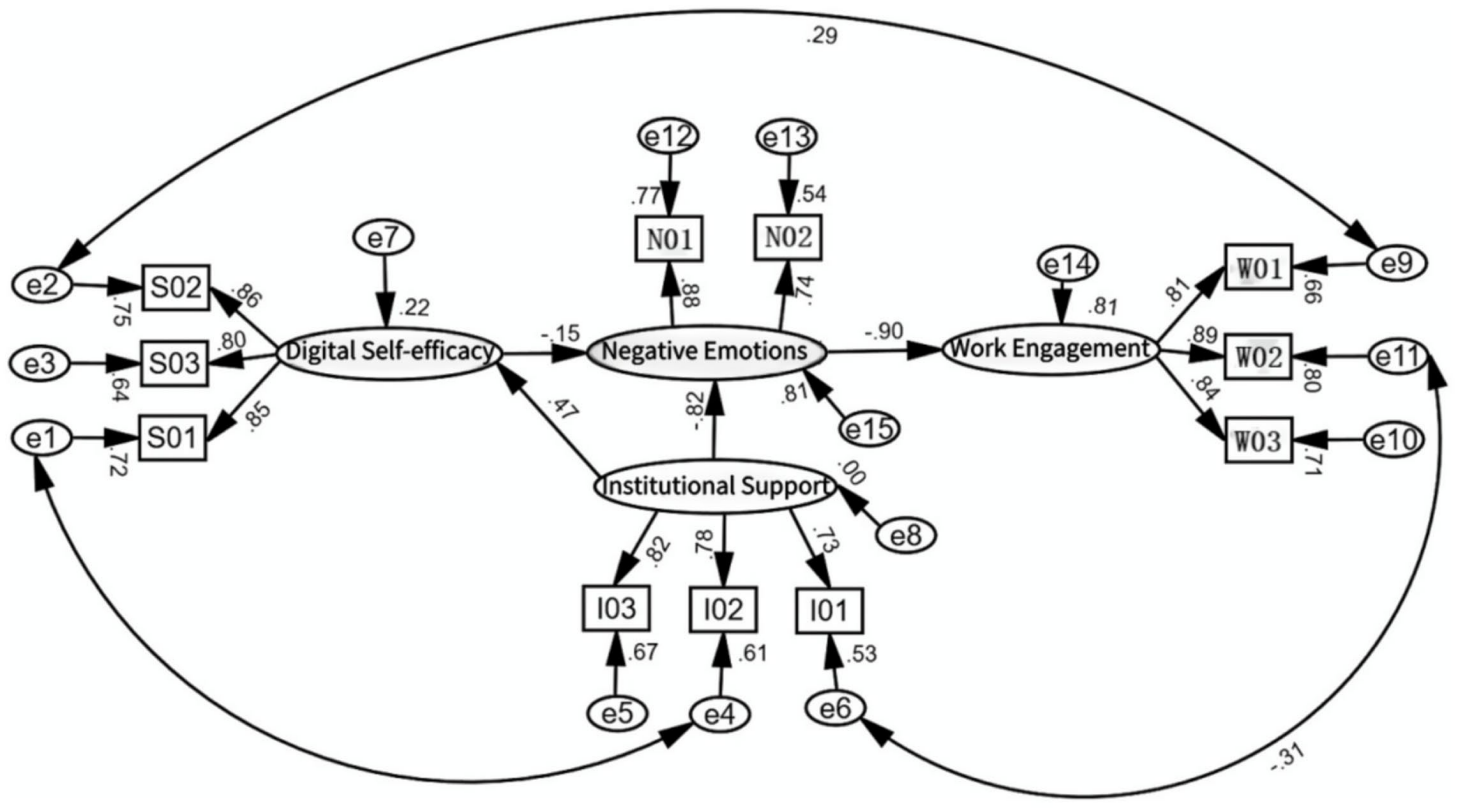
The final structural model (standardized parameter estimates).

The indices of the final model revealed that the model fit the data well. The fit indexes of the revised structural equation model are shown in Table 3, and the final structural model in Figure 3. The results showed that institutional support significantly predicted teachers’ digital self-efficacy (*β* = 0.47, *p* < 0.01) and negative emotions (*β* = −0.82, *p* < 0.01). Digital self-efficacy significantly predicted negative emotions (*β* = 0.15, *p* < 0.01). Teachers’ negative emotions significantly predicted work engagement (*β* = −0.90, *p* < 0.01). Therefore, H1b, H1c, H2a, and H3b are valid and H1a and H3a are not valid.

**Table 3:** Final structural model fit index.

	χ2 /df	AGFI	GFI	NFI	CFI	IFI	RMSEA
Values	2.306	0.897	0.942	0.949	0.970	0.971	0.075

### 4.3 Mediated relations

**Figure 4:**
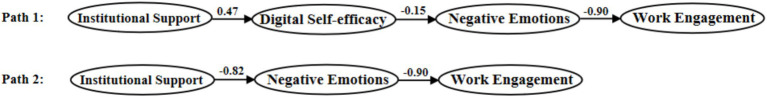
The two chain mediation effects.

The study conducted a mediation effects test through Bootstrap 5,000 times in AMOS 26.0. As shown in Table 4 and Figure 4, this study supports two chain mediation effects.

**Table 4:** Chain mediation effects.

Paths	Estimate	S.E.	*p*	Bias-corrected percentile bootstrap method	
				Upper bound of the 95% CI	Lower bound of the 95% CI
1: Institutional support → Negative emotions → Work engagement	0.913	0.103	0.000	1.132	0.726
2: Institutional support → Digital self-efficacy → Negative emotions → Work engagement	0.080	0.038	0.023	0.166	0.011
Total effects	0.993	0.098	0.000	1.198	0.810
Path comparison	−0.832	0.121	0.000	−0.612	−1.083

Path 1: Institutional support → Negative emotions → Work engagement.

BootstrapCI 95% of the mediating effect of online teaching negative emotions did not contain 0 [0.726, 1.132]. And the effect value was 0.913 accounting for 91.94% of the total effects.

Path 2: Institutional support → digital self-efficacy → negative emotions → work engagement.

BootstrapCI 95% of the chain mediation effects of digital self-efficacy and negative emotions did not contain 0 [0.011, 0.166], with an effect value of 0.080, or 8.06% of the total effects.

Therefore, H2b and H3d were valid. And H3c was not valid. In addition, the Bootstrap CI 95% for the comparison of the two paths was [0.810, 1.198], respectively, neither of which contained 0. It indicated a significant difference in the chain mediation effects of the two paths.

### 4.4 Influence of gender

An independent samples t-test was conducted to investigate whether gender was related to institutional support, digital self-efficacy, negative emotions and work engagement. The results showed that there was a significant difference between genders in institutional support (*p* = 0.002 < 0.05) and work engagement (*p* = 0.011 < 0.05). Specifically, female teachers perceived more institutional support for online teaching than male teachers. And female teachers had higher work engagement in online teaching than male teachers.

### 4.5 Influence of age

ANOVA was conducted to investigate whether age was related to the four dimensions. Teachers between the ages of 20–29 and 30–39 had significant differences in negative emotions (*p* = 0.002 < 0.05), institutional support (*p* < 0.001) and work engagement (*p* = 0.001 < 0.05). Specifically, 20–29 year old teachers experienced less negative emotions than 30–39 year old teachers, but better than 30–39 year old teachers in perceiving of institutional support, and work engagement. In addition, there was a significant difference between teachers aged 20–29 and 40–49 in terms of negative emotions (*p* = 0.026 < 0.05), digital self-efficacy (*p* = 0.010 < 0.05), and institutional support (*p* < 0.001). Specifically, teachers aged 20–29 experienced fewer negative emotions than teachers aged 40–49, and were better in digital self-efficacy, and perceiving institutional support than 40–49 teachers. There was a significant difference between teachers aged 30–39 and 50 + in negative emotions (*p* = 0.003 < 0.05), and institutional support (*p* < 0.001). Specifically, teachers aged 50 experienced less negative emotions than teachers aged 30–39, and better than teachers aged 30–39 in perceiving of institutional support. There was a significant difference between teachers aged 40–49 and 50 + in negative online emotions (*p* = 0.003 < 0.05), institutional support (*p* < 0.001), and work engagement (*p* = 0.005 < 0.05). Particularly, teachers aged 50 + experienced less negative emotions than teachers aged 40–49, and were better in perceiving institutional support, and online teaching engagement than teachers aged 40–49 teachers. In addition, there was no significant difference between 20 and 29 and 50+, 30–39, and 40–49-year-old teachers in the four dimensions.

### 4.6 Influence of teaching years

ANOVA was conducted to investigate whether teaching years were related to the four dimensions. The results showed that there was a significant difference between teachers with less than 5 years of teaching experience and those with 6–10 years of institutional support (*p* = 0.007 < 0.05). Specifically, teachers with less than 5 teaching years were better than teachers with 6–10 teaching years in perceiving of institutional support. And there was a significant difference between teachers with less than 5 years and teachers with more than 10 teaching years in negative emotions (*p* = 0.013 < 0.05), digital self-efficacy (*p* = 0.003 < 0.05), institutional support (*p* < 0.001), and work engagement (*p* = 0.001 < 0.05). In particular, teachers with less than 5 teaching years had less negative emotions than those with more than 10 teaching years, and were higher in digital self-efficacy, perceiving innovation support and work engagement than teachers with more than 10 teaching years. In addition, there were no significant differences between teachers with more than 5 teaching years and teachers with more than 10 teaching years on the four dimensions.

### 4.7 Influence of education level

ANOVA was conducted to investigate whether education level had a significant influence on the four dimensions. In terms of teachers’ education level, all the participants had an associate degree or above. There was a significant difference between teachers with undergraduate degrees and those with graduate degrees in negative emotions (*p* = 0.002 < 0.05), digital self-efficacy (*p* = 0.035 < 0.05), institution support (*p* = 0.002 < 0.05), and work engagement (*p* = 0.002 < 0.05). Specifically, teachers with an undergraduate degree experienced less negative emotion in online teaching than teachers with a graduate degree, and also higher in digital self-efficacy, perceiving innovation support and work engagement than teachers with a graduate degree. Additionally, there was no significant difference in the four dimensions between teachers with associate degrees and teachers with undergraduate degree or graduate degree.

## 5 Discussion

This study found indirect effects of institutional support on work engagement in online teaching. The independent mediating effect of negative emotion on the relationship between work institutional support on work engagement was significant. The results indicated a significant chain mediation effect, where the mediator digital self-efficacy affected the mediator negative emotion, which subsequently influenced the work engagement in online teaching.

### 5.1 Negative emotions as mediator

The study found that online teaching negative emotions mediated the relationship between institutional support and work engagement in online teaching. Institutional support supports teachers not only with substantive technical support, but also with psychological support. It was in order to help teachers reduce negative emotions associated with online teaching by relieving the stress of technology use. The finding was consistent with previous researches (Joo et al., 2016; Vongkulluksn et al., 2018). Joo et al. (2016) emphasized institutional support’s dual role in providing technical resources (e.g., reliable platforms, IT assistance) and psychological reassurance (e.g., peer mentoring, stress management workshops). For instance, technology-specific anxiety is mitigated when schools offer real-time technical hotlines, while burnout is alleviated through workload redistribution policies (Vongkulluksn et al., 2018). Unlike anxiety, which often arises from transient technical challenges, anger is more deeply rooted in systemic inequities or perceived institutional neglect. For example, teachers reported anger when forced to adopt poorly tested technologies without consultation, echoing Ding (2025) observation that anger emerges when autonomy is undermined. In addition, teachers can conduct online teaching with a positive attitude through the reduction of negative emotions of online teaching, resulting in increased work engagement in online teaching. Moreira-Fontán et al. (2019) demonstrated that ICT positive emotions mediate the relationship between innovation support of ICT and work engagement, which was consistent with the findings of this study.

The differences between the two mediating effect paths in this study were found to be significant. And the mediating effect of negative emotions is much higher than the chain mediating effect of digital self-efficacy and negative emotions. It suggests that negative emotions were more relevant in online teaching. A study conducted by Azzaro and Martínez Agudo (2018) showed that emotions play an important role in teaching environment where technology is used. Therefore, the impact of emotions in online teaching environments needs to be paid more attention.

The three strategies are proposed based on the findings. First, school administrators have a decisive influence on teachers’ intentions to use technology for learning and teaching (Instefjord and Munthe, 2017; Teo and Noyes, 2011). And they should develop mechanisms to motivate teachers in order to be more positive in online teaching. Second, schools should provide more opportunities for teachers to communicate, as effective communication can also contribute to a positive working environment which helps teachers to work with greater ease and pleasure. For example, establish transparent communication channels for teacher feedback during technology rollout and ensure equitable workload distribution (Giray, 2025). Third, deploying AI-Driven Emotional Support Tools (AI Chatbots et al.) trained on cognitive-behavioral therapy (CBT) principles to reduce teacher workloads. Furthermore, other negative emotions such as frustration and burnout can also affect teachers’ engagement in online teaching (Aldahdouh et al., 2024). Strategies to reduce teachers’ frustration and burnout will be examined in future research.

### 5.2 Digital self-efficacy as mediator

According to the JD-R theory, job resources and personal resources may support each other and reduce stress caused by work demands. Institutional support is a job resource. Institutional support is a type of job resource. Digital self-efficacy is a type of personal resource. Emotions, such as job-related anxiety, are also sources of stress. The JD-R confirms the negative effects of digital self-efficacy and institutional support on negative emotions in online teaching.

Online teaching is a new form of teaching different from traditional classroom teaching. Online teaching is a novel way of teaching that differs from traditional classroom teaching. To adapt to the new job requirements, teachers must make adjustments to their instructional resources, methods, and other aspects. Teachers would have negative emotions when switching from their previous teaching mode. Institutional support and teachers’ digital self-efficacy can help reduce negative emotions and improve work engagement. It is consistent with the findings of Xanthopoulou et al. (2009), who discovered that job resources predict personal resources (self-efficacy) and work engagement.

The four evidence-based strategies were proposed. First, institution could establish regional hubs for sharing digital teaching resources and support strategies, mirroring Japan’s GIGA School Program (MEXT, 2022), which provides uniform devices and cloud-based lesson templates, minimizing variability in institutional support (Tanaka and Yamada, 2023). Second, school administrators are required to develop a teacher professional development plan to enable the successful integration of information technology and education that includes an online instructional plan (Vongkulluksn et al., 2018). In Romania, the central administration of education contributes to the development of teachers’ competences through their participation in continuous training programs (Tripon, 2022). And certification systems should be advocated that standardize digital teaching skills while integrating emotional resilience training. In addition, certification should be linked to career advancement. Third, Teachers must also conduct a learning session on an online course instructional approach and extensively complete the instructional design of the online courses. In fact, teachers have a high demand for training in online teaching knowledge and competency. Song et al. (2020) proposed that more than 70% of teachers need to be trained in the use of platforms and access to digital resources. Fourth, establish observation groups where exemplary teachers share recorded lessons and problem-solve collaboratively. According to Song et al., another strategy to improve digital self-efficacy is to observe other teachers who use information technology well in the classroom. In practice, teachers who are more effective in online teaching should be encouraged to record their online teaching for other teachers to reference and learn from. Each group should include excellent teachers to share their successful online teaching experience. The members share their learning experiences and discuss the problems related to online teaching. It helps to improve their work engagement and online teaching effectiveness.

### 5.3 Influence of teacher work engagement

In this study, teacher work engagement was investigated as an outcome variable. But it can in turn actively moderate the relationship between digital self-efficacy and negative emotions. The influence is reflected in two aspects.

First, high engagement should be buffer low digital self-efficacy. Teachers with high work engagement exhibited lower levels of negative emotions even when their digital self-efficacy was moderate or low. It aligns with the buffering hypothesis within the JD-R framework (Bakker and Demerouti, 2017), where engagement—a personal resource—mitigates the detrimental effects of limited self-efficacy. Specifically, teachers with high engagement reframed technological challenges as opportunities for pedagogical innovation, thereby reducing frustration and anxiety (Schaufeli, 2015). The adaptive response pattern corresponds with the transactional stress model (Lazarus and Folkman, 1984), suggesting that engagement facilitates the development of constructive coping strategies.

Second, low engagement exacerbated negative emotions triggered by low self-efficacy. Teachers with reduced engagement levels were more likely to attribute technical failures to personal inadequacy (e.g., “I lack inherent technical competence”) rather than situational constraints, intensifying emotions of anger and burnout (Bandura, 1997). Crucially, sustained engagement appears to foster self-efficacy through two complementary pathways: (1) mastery experiences derived from overcoming technical challenges (e.g., resolving live-streaming disruptions), and (2) vicarious learning through peer observation (e.g., adopting colleagues’ successful digital strategies), collectively disrupting negative emotional cycles (Salanova et al., 2016).

### 5.4 Innovative practices on online teaching platform

Within the landscape of Chinese online education, platforms such as Tencent Meeting and DingTalk have emerged as dominant tools. The participants in this study have experience teaching online on Tencent Meeting or DingTalk (China Internet Network Information Center, 2020). These platforms exemplify how institutional support intersects with pedagogical innovation, shaping teachers’ digital self-efficacy and emotional experiences. Based on the online teaching platforms, three types of digital teaching practices were proposed.

Type 1, Innovative Practices for Synchronized Interaction. Tencent Meeting, widely adopted for synchronous instruction, facilitates real-time interactive teaching through features like breakout rooms for small-group collaboration, shared digital whiteboards for co-constructing knowledge. These tools reduce frustration by enabling dynamic student-teacher interactions, but require institutional training to mitigate technology-specific anxiety (Wang and Li, 2022). In the future, it is important to continue to optimize and use smart technology in online teaching. Design targeted training based on teachers’ instructional needs and create dynamic learning programs to reduce technology anxiety (Wang and Li, 2022).

Type 2, Innovative Practices for asynchronous adaptation. Conversely, excels in supporting asynchronous adaptation via its “Smart Homework” system, which automates grading and generates personalized feedback using optical character recognition (OCR) and machine learning. Such practices enhance digital self-efficacy by providing data-driven insights (e.g., learning analytics dashboards) (Chen M. et al., 2022), yet may provoke negative emotion if workload balancing mechanisms are absent (Li et al., 2024). Therefore, future strategies should prioritize enhancing personalization through multimodal data integration (text, voice, video) while expanding analytics dashboards with real-time emotional feedback mechanisms to improve teacher digital self-efficacy (Chen I. H. et al., 2022; Li et al., 2024).

Type 3, innovative practices for hybrid pedagogical models. Tencent Meeting’s API compatibility allows seamless embedding of third-party tools like VR science labs, while DingTalk’s “DingTalk Classroom” combines live streaming with asynchronous discussion boards and cloud-based resource libraries. Teachers often hybridize these platforms: hosting synchronous lectures on Tencent Meeting, then assigning DingTalk’s AI-graded reflections to consolidate learning. The hybrid pedagogical model foster engagement through creative autonomy but demand robust institutional infrastructure to prevent anger from systemic inequities (Hill and Reimer, 2023).

## 6 Conclusions and implications

Compared to traditional learning environments, online teaching and learning environments differ in form, function, characteristics and patterns (Nygren et al., 2019). Consequently, there have been significant changes to the associated processes of teaching and learning as well as the methods of producing, sharing, and gaining access to educational materials. Numerous new issues have resulted from this, including how teachers should adopt appropriate learning strategies and optimize the structure of their teaching approaches (Wang et al., 2024). This study contributes to research on teacher work engagement in online teaching in three ways. First, the study hypotheses were proposed based on the previous theory. Second, the validated questionnaire was applied to empirical research. And the study confirms that digital self-efficacy significantly predicted negative emotions, which was predicted work engagement, as well as online teaching negative emotions and digital self-efficacy mediated the relationship between institutional support and work engagement in online teaching. In addition, gender, age, teaching years, and education level were related to variables. Third, based on these findings, the relationships between variables were discussed. And strategies were put forward to improve teacher work engagement in online teaching.

This study has theoretical implications in that it contributes to online teaching theory. The hypotheses explained the relations between variables. A practical implication is that this study offers a reference for teachers and administrators in online teaching systems. From a practical perspective, digital self-efficacy and negative emotions could be modified. And they are highly dependent on different degrees of institutional support. Administrators and educational institutions should offer sufficient initial training as well as efficient and continuous professional development programs to ensure that pre-service and in-service teachers successfully integrate digital technologies into their teaching. This will allow them to capitalize on the impact of institutional support in enhancing digital self-efficacy and reducing negative emotions (Teo et al., 2016). Moreover, educational institutions such as schools should measure the level of knowledge and competence of teachers about online teaching. According to the results of the measurement and the needs of teachers, the institutions should also design different contents and forms of training and provide teachers with stratified training in order to finally improve teacher work engagement in online teaching.

## 7 Limitations and future research

One of limitation is that only four latent variables were measured. The four variables include both individual factors and organizational factors. However, other factors, such as motivation, positive emotion, might also have an effect on work engagement in online teaching. In future research, a complex model with more correlated variables should be proposed, and more comprehensive suggestions should be put forward. Another limitation is only quantitative methods were applied in this study. In the future, qualitative methods including classroom observation and interviews should be adopted in order to analyze the complex causes of the formation of the phenomenon. Finally, the sample lacks diversity. The sample for the study was concentrated in the eastern and northern parts of China, but the overall number and representation of the sample are in need of further consideration. In future research, a larger sample including teachers from other areas will be used, to put forward more general conclusions. Furthermore, the findings of this study suggest that institutional support enhances teachers’ engagement to adopt innovative practices, which in turn may improve instructional effectiveness. To investigate this relationship, future research should assess the effectiveness of online teaching through the students’ academic performance dimension, the teacher’s psychological and behavioral dimension.

## Data Availability

The raw data supporting the conclusions of this article will be made available by the authors, without undue reservation.
